# The neural processing of hierarchical structure in music and speech at different timescales

**DOI:** 10.3389/fnins.2015.00157

**Published:** 2015-05-12

**Authors:** Morwaread M. Farbood, David J. Heeger, Gary Marcus, Uri Hasson, Yulia Lerner

**Affiliations:** ^1^Department of Music and Performing Arts Professions, New York UniversityNew York, NY, USA; ^2^Department of Psychology and Center for Neural Science, New York UniversityNew York, NY, USA; ^3^Department of Psychology, New York UniversityNew York, NY, USA; ^4^Department of Psychology and the Neuroscience Institute, Princeton UniversityPrinceton, NJ, USA; ^5^Department of Neurology and the Functional Brain Center, Tel Aviv Sourasky Medical CenterTel Aviv, Israel; ^6^Sackler Faculty of Medicine, Tel Aviv UniversityTel Aviv, Israel

**Keywords:** music, speech, processing timescales, hierarchical structure, fMRI

## Abstract

Music, like speech, is a complex auditory signal that contains structures at multiple timescales, and as such is a potentially powerful entry point into the question of how the brain integrates complex streams of information. Using an experimental design modeled after previous studies that used scrambled versions of a spoken story (Lerner et al., 2011) and a silent movie (Hasson et al., 2008), we investigate whether listeners perceive hierarchical structure in music beyond short (~6 s) time windows and whether there is cortical overlap between music and language processing at multiple timescales. Experienced pianists were presented with an extended musical excerpt scrambled at multiple timescales—by measure, phrase, and section—while measuring brain activity with functional magnetic resonance imaging (fMRI). The reliability of evoked activity, as quantified by inter-subject correlation of the fMRI responses, was measured. We found that response reliability depended systematically on musical structure coherence, revealing a topographically organized hierarchy of processing timescales. Early auditory areas (at the bottom of the hierarchy) responded reliably in all conditions. For brain areas at the top of the hierarchy, the original (unscrambled) excerpt evoked more reliable responses than any of the scrambled excerpts, indicating that these brain areas process long-timescale musical structures, on the order of minutes. The topography of processing timescales was analogous with that reported previously for speech, but the timescale gradients for music and speech overlapped with one another only partially, suggesting that temporally analogous structures—words/measures, sentences/musical phrases, paragraph/sections—are processed separately.

## Introduction

To understand language, the brain must integrate information across a broad range of timescales, from tens of millisecond (e.g., words), to seconds (e.g., sentences), and even minutes (discourse). Composers often construct pieces that vary over similar durations, yet it has often been suggested that music, unlike language, might be analyzed at only brief timescales of up to a few seconds. Several behavioral studies, for instance, have indicated that musical events happening outside of that short time span have little bearing on what is perceived in the moment (Levinson, 1997; Tillmann et al., 1998; Bigand and Parncutt, 1999; Tillmann and Bigand, 2004); others have suggested that listeners are not very sensitive to music scrambled at phrase-length timescales, and that scrambling has a limited impact on perception of tonal coherence (Karno and Konečni, 1992; Tillmann and Bigand, 1996; Marvin and Brinkman, 1999; Lalitte and Bigand, 2006; Eitan and Granot, 2008; Granot and Jacoby, 2011). However, these prior studies, as well as many neuroimaging and event-related potential (ERP) studies comparing music and language (Besson and Schön, 2001; Maess et al., 2001; Patel, 2003; Koelsch et al., 2005, 2013; Carrus et al., 2011), have focused primarily on tonality, possibly at the expense of other important structural elements in music such as melody and texture. This is most likely due to two convergent factors: the central importance of tonality in Western music theory and the natural comparisons that can be drawn between tonality and syntax (cf. Lerdahl and Jackendoff, 1983).

Comparisons between tonality and syntax have played a significant role in research that has examined possible overlaps between music and language processing. According to the influential shared syntactic integration resource hypothesis (Patel, 2003), long-term knowledge about the structure of music is stored separately and independently from that for language, but the system used for “online structural integration” (corresponding roughly to the working memory processes needed for syntactic parsing) may be shared (Fedorenko et al., 2009). Despite the apparent similarities between music and speech—they are both complex, highly structured auditory signals—there are also significant differences. Direct communication is the primary purpose of language, while musical structures do not have semantic content (Slevc and Patel, 2011). If it is the case that listeners cannot apprehend musical structures over long time spans, the inherent lack of precise, explicit meaning in music could be one possible factor. Furthermore, the building blocks of speech are encoded primarily through timbral changes as opposed to discrete pitch changes (Zatorre et al., 2002; Patel, 2008).

The current study addresses two open questions: first, to what extent do listeners make structural connections over longer (>5–8 s) timescales in music? Second, to what extent does temporal processing of music and speech—at both short and long timescales—overlap? Related to the second point is the question of what types of structural comparisons between components of speech and music are appropriate, assuming such analogous structures exist between the two domains (e.g., phrases in music versus phrases/sentences in speech). Using functional magnetic resonance imaging (fMRI), we investigated the extent to which expert listeners process musical structure over long timescales and the extent to which temporal processing of music and speech overlap at different timescales corresponding to formal structures. In contrast to a few previous studies that explored brain responses to free listening of music over long time periods without regard to structure (Alluri et al., 2011; Abrams et al., 2013), our explicit emphasis was on *hierarchical* differences. By hierarchical structure we refer specifically to the concept of *form* that is central to Western music theory (Koch, 1983; Bent, 1994; Marx, 1997), the principal levels of structure typical in the analysis of form being sections and phrases (Rosen, 1988; Caplin, 1998; Hepokoski and Darcy, 2006).

Our experimental protocol was modeled after previous studies that used scrambled versions of a spoken story (Lerner et al., 2011) and a silent movie (Hasson et al., 2008). Lerner et al. (2011) measured the reliability of evoked activity through inter-subject correlation (inter-SC) of the BOLD response time courses. Brain activity was recorded as participants listened to a 7-min spoken story as well as scrambled versions of the original story segmented by word, sentence, and paragraph. In addition, subjects also listened to a reversed, or backwards version of the original waveform. Their results revealed that brain responses to a narrated story encompass a nested hierarchy for temporal processing at different timescales. Note that such hierarchical organization of human speech perception might reflect the organization of the auditory pathways in other species as well. For example, a hierarchical organization based on the complexity of the auditory sounds, from single tones and noise toward more complex ecological sounds, has been demonstrated in the auditory system of bats (Suga et al., 1979), songbirds (Margoliash and Fortune, 1992), and non-human primates (Rauschecker et al., 1995). Specifically, in non-human primates, a topographic organization has been observed in which lower-order neurons are responsive to relatively simple features and higher level neurons and local networks are selective for increasingly complex auditory stimuli (Poremba et al., 2003; Bendor and Wang, 2005; Rauschecker and Scott, 2009).

Analogous to Lerner et al. (2011), we manipulated musical content by scrambling a musical excerpt at different temporal-structural levels, and measured response reliability in the brains of experienced pianists. We designed our music study so that the fMRI experiment parameters were nearly the same as those used in the Lerner et al. story study—similar population size, same MRI equipment, and nearly identical scanning protocol. This design allowed us to investigate the possible cortical overlap between music and language processing at multiple timescales by directly comparing the data from the Lerner et al. story experiment to our music data. Unlike prior work examining brain response to scrambled music and/or speech (Levitin and Menon, 2003; Abrams et al., 2011, 2013; Rogalsky et al., 2011), we used timescales that encompassed all levels of musical form.

## Methods

### Participants

Twenty-five experienced pianists participated in the fMRI study, recruited from the Juilliard piano performance program and the New York University Department of Music and Performing Arts Professions piano and jazz performance programs. None of the participants took part in the earlier Lerner et al. (2011) story experiment. One participant was excluded from the analyses due to anatomical abnormalities. Runs in which head motions were greater than 2 mm were discarded from the analyses, as were runs in which the signal was corrupted by obvious artifacts (e.g., spikes in the fMRI time series greater than a five standard deviation change in image intensity), and runs in which the slice prescriptions did not completely cover the brain areas of interest. Participants were recruited until we had acquired usable data (according to the above criteria) for 15 runs per experimental condition. More than 15 participants were needed because some individual runs were excluded, resulting in incomplete sets of runs. A total of 18 participants with complete or partial sets of usable data were included in the analysis. All participants (7 female, 11 male, all right-handed except for one participant, *M* = 23.78 years of age, *SD* = 6.44) had significant experience in either jazz or classical piano performance (*M* = 16.00 years of piano instruction, *SD* = 3.92), and practiced regularly (*M* = 11.28 h per week, *SD* = 7.80). All except one listed classical music as a listening preference, and 6 reported having absolute pitch. The experimental procedures were approved by the University Committee on Activities Involving Human Subjects (UCAIHS) at NYU, and all participants provided written informed consent.

### Stimuli

Stimuli were generated from a musical excerpt, the first 4′15″ of the third movement of Brahms Piano Concerto No. 1 in D minor performed by pianist Krystian Zimerman and the Berlin Philharmonic conducted by Simon Rattle. Participants rated their familiarity with the piece on scale from 1 to 5 where 1 = completely unfamiliar and 5 = very familiar, resulting in a self-reported mean familiarity rating of 2.39, *SD* = 1.42. None of the participants had performed the piece, although one had practiced it; 7 reported being completely unfamiliar with the piece.

**Figure 1:**
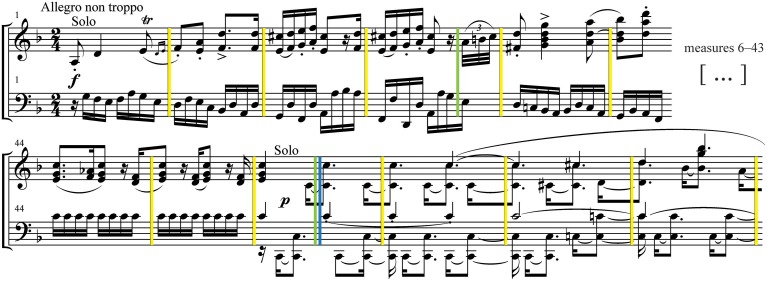
**Stimuli**. A schematic representation of the stimuli used in the experiment. The score of the third movement of Brahms Piano Concerto No. 1 in D minor was segmented at multiple timescales defined by measures (yellow), phrases (green), and sections (blue). The digital audio recording was segmented at the corresponding time points, and then reassembled with the segments in randomly shuffled order.

Modified versions of the excerpt were all derived from the original audio recording (“intact” version) scrambled at three timescales, by segmenting the original excerpt at measure, phrase, and section boundaries, and then randomly reassembling the audio segments for each condition (Figure 1). The version scrambled at the shortest timescale (“measures”) consisted of 193 measures (*M* = 1.29 s, *SD* = 0.12); the intermediate scale (“phrases”) consisted of 40 phrases (*M* = 6.32 s, *SD* = 1.91); and the longest scale (“sections”) consisted of 7 sections (*M* = 38.28 s, *SD* = 12.46). Additionally, a “backward” version—the time-reversed waveform of the original audio—was generated.

The segmentation boundaries for the scrambled stimuli were first determined through analysis of the musical score. The analysis was done by the first author (who has extensive training in composition and theory) and followed general music-theoretic guidelines. The Brahms excerpt was chosen in part because it was easy to parse and the segmentation boundaries were relatively clear. The boundary time points in the audio were then determined manually by ear within a 5–10 ms window using the audio editing program Audacity (v. 2.0). Establishing these precise times was somewhat difficult due to reverberation in the recording and the soft attacks of the orchestra that were often unsynchronized with the sharp attacks of the piano. Once the best segmentation time points were determined, they were used as input to an audio-scrambling Python script written with the Echo Nest Remix API 3.0 (Jehan, 2010). The script randomly shuffled the audio segments at the designated boundaries, adding 10 ms crossfades between each segment to eliminate clicks. The randomization algorithm ensured that no two consecutive segments ended up in sequential order by accident. After these new scrambled versions were generated, dynamic range compression was applied to the audio files (using the Compressor effect in Audacity) to reduce the loudness contrasts between the rearranged segments. This helped reduce jarring dynamic changes and increased the relative loudness of softer sections to be more audible in the scanner.

### Procedure

#### Main experiment

All conditions except the backward stimulus were presented twice over the course of a typical scanning session. A typical scanning session consisted of two sets (Run 1 and Run 2), each comprising the presentation of intact, measures, phrases, and sections conditions in a pseudo-randomized order. The stimuli for each condition were identical across participants and across the two runs for each participant. The original excerpt was always presented as the first stimulus in the first set, and the last stimulus in the second set. The scrambled versions were presented in different order, counterbalanced between participants. The backward condition was presented at the very end, following the second set. Participants were instructed to listen attentively to the stimuli. Three seconds of silence preceded playback of each condition; fMRI data acquired during these silent periods were discarded from the analyses.

#### MRI acquisition

MRI scanning was carried out at the NYU Center for Brain Imaging, with a 3T head-only MRI scanner (Allegra; Siemens, Erlangen, Germany), using a custom radio frequency transmit/receive head coil (NM-011; NOVA Medical, Wakefield, MA). Blood oxygenation level dependent (BOLD) functional MRI was acquired with *T*2^*^-weighted, gradient recalled echo-planar imaging: repetition time (TR) = 1500 ms; echo time (TE) = 30 ms; flip angle = 75°; 172 volumes per run; 26 slices; slice thickness = 3 mm thickness, 1 mm gap, in-plane resolution = 3 × 3 mm. The slices were positioned nearly horizontal, tipped slightly forward (with the lower part at the front) to get the entire temporal lobe and the parts of the frontal lobe that are involved in hearing and language processing as well as nearly all of the occipital and parietal lobes. After the music listening part of the experiment, a high-resolution anatomical volume was acquired for each participant using a magnetization-prepared rapid gradient echo (MP-RAGE) *T1*-weighted sequence: *TR* = 2500 ms; *TE* = 4 ms; 176 slices; slice thickness = 1 mm, no gap; in-plane resolution = 1 × 1 mm; in-plane field of view = 256 × 256 mm. This anatomical volume was used for cortical segmentation and surface reconstruction. To minimize head movements, participants' heads were stabilized with foam padding. Stimuli were presented using Psychtoolbox for MATLAB (Brainard, 1997; Pelli, 1997; Kleiner et al., 2007). Sensimetrics insert earphones were fitted underneath MR Confon Optime 1 headphone noise guards to present the audio stimuli and provide considerable attenuation of the scanner noise.

### Data analysis

#### Preprocessing

Neuroimaging data were analyzed using BrainVoyager QX software (Brain Innovation, Maastricht, Netherlands) and with additional software written in MATLAB. Preprocessing of the functional data consisted of slice time and motion correction, linear trend removal, high-pass filtering (cut-off: 0.01 Hz), spatial smoothing with a Gaussian filter (6 mm full-width at half-maximum value), and cropping of the first 15 TRs in each run to allow the hemodynamic responses to reach steady state. The cortical surface was reconstructed from anatomical images using standard procedures implemented in the BrainVoyager QX software. The complete functional dataset was transformed to Talairach coordinates (Talairach and Tournoux, 1988) and projected on an inflated reconstruction of the cortical surface.

#### Inter-subject correlation analysis

Data were analyzed using inter-SC analysis, which measures the reliability of the responses to natural stimuli by comparing the fMRI responses across participants (Hasson et al., 2010). Correlation maps were constructed on a voxel-by-voxel basis (in Talairach space), separately for each condition (intact, backward, measures, phrases, and sections), by comparing the fMRI response time courses across listeners. First, the Pearson product-moment correlation *r_j_* = *corr*(*TC_j_*, *TC*_*All−j*_) was computed between a voxel's fMRI time course *TC_j_* in one individual and the average *TC*_*All−j*_ of that voxel's fMRI time courses in the remaining participants. Next, the average correlation $R=\frac{1}{N}\sum_{j=1}^{N}r_{j}$ was calculated at every voxel. The analysis revealed systematically stronger correlations within Run 1 than Run 2, so Run 1 was used in all further analyses.

#### Statistics

Statistical significance of inter-SCs was assessed using a phase-randomization procedure. Phase-randomization was performed by applying a fast Fourier transform to the signal, randomizing the phase of each Fourier component, and then inverting the Fourier transformation. Thus, the power spectrum was preserved but the correlation between any pair of such phase-randomized time courses had an expected value of 0. Phase-randomized time courses were generated for every measured fMRI time course from every voxel in each participant. A correlation value was then computed (as detailed above) for every voxel. This process was repeated 5000 times to generate a null distribution of the correlation values, separately for each voxel. Statistical significance was assessed by comparing empirical correlation values (without phase randomization) with these null distributions. The Benjamini–Hochberg–Yekutieli false-discovery procedure, which controls the false discovery rate (FDR) under assumptions of dependence, was used to correct for multiple comparisons (Benjamini and Hochberg, 1995; Benjamini and Yekutieli, 2001; Genovese et al., 2002). Specifically, *p*-values were sorted in ascending order and the value p_q^*^_ was chosen as the *p*-value corresponding to the maximum *k* such that *p_k_* < $\frac{k}{N}$q^*^, where q^*^ = 0.05 is the FDR threshold, and *N* is the total number of voxels.

#### Temporal receptive window maps for music

Previous studies have shown that the processing timescale increases from low-level sensory areas to high-level frontal and parietal areas (Hasson et al., 2008; Lerner et al., 2011; Honey et al., 2012). By analogy with the notion of a spatial receptive field, the temporal receptive window (TRW) of a neural circuit can be defined as the length of time prior to a response during which sensory information may affect that response. TRWs are short in sensory areas, and become gradually longer toward higher-order areas. To characterize the TRWs within each brain area, we parametrically varied the temporal structure of an extended musical excerpt by breaking it into smaller and smaller temporal units (section, phrase, measure) and then scrambling the segments, as described above. Next we asked whether the responses to each event changed as a function of prior events. Areas with short TRWs were expected to respond in the same way to each event regardless of the temporal coherency of the music. Areas with long TRWs were expected to modulate their responses to a given event as a function of the temporal coherency of the extended musical excerpt over many seconds. Following Lerner et al. (2011) and Hasson et al. (2008), we constructed a nested map of TRWs, classifying the voxels according to the shortest temporal structure that evoked reliable responses.

#### Analyses comparing music and story data

In addition to analyzing the music data by scrambling conditions, we ran a series of analyses that compared the music data to the story data from Lerner et al. (2011). We considered running the story experiment on our current expert musician group but chose not to do so in part because there was little reason to believe that processing of speech at different timescales varies as a function of musical expertise. Regarding differences in musical structure processing between musicians and nonmusicians, a caveat is necessary: it might be that these findings are specific to professional musicians, who have significantly enhanced sensorimotor processing ability than the general population. It is possible that the hierarchical topography of music processing may follow a different, perhaps more simplistic pattern in musically untrained listeners. On the other hand, there is considerable evidence showing that nonmusicians, through listening alone, acquire the capacity to understand musical structure to a degree of sophistication that enables them to respond to music in much the same way musicians do (Bigand and Poulin-Charronnat, 2006).

The music-story analysis consisted of two parts: first, responses to the intact music from the current study and the responses to the intact story condition from the Lerner et al. (2011) study were compared to determine where reliable responses in both cases overlapped. Next, ROIs defined along the temporo-parietal axis based on results from both the music and story experiments were examined. An early auditory ROI (A1+) was defined as the set of voxels that correlated the most with the stimulus audio envelope. To compute the correlation between BOLD signals and the audio envelope, we bandpass filtered the audio signal between 4 and 4000 Hz, extracted the envelope of the signal using a Hilbert transform, and then downsampled the envelope to the sampling rate of the BOLD signal using an anti-aliasing lowpass finite impulse response filter.

The other ROIs were defined as a sequence extending from A1+ to higher-order areas of auditory cortex posteriorly along the temporo-parietal axis. To sample the responses without bias, we defined two axes in the left hemisphere (for both music and story) and another in the right hemisphere (for music only). The story axis was defined manually within the extent of reliable responses to the intact story condition (**Figure 4**, right map, *q* < 0.05, FDR corrected). The defined axis was then used for analyses of responses induced by story (top plots) and music (bottom plots) stimuli. For convenience, the defined story axis is shown on the map of responses to the intact music condition (**Figure 4**, left map). Analogously, the music axes were defined within the extent of reliable responses to the intact music condition (**Figures 5, 6**, left maps, *q* < 0.05, FDR corrected). The ROIs were evenly spaced along each of these axes by manually partitioning the volume into adjacent, cubic sub-regions of approximately equal size.

## Results

### Inter-subject correlation analysis in the cortical regions

#### Responses to different music scrambling conditions

**Figure 2:**
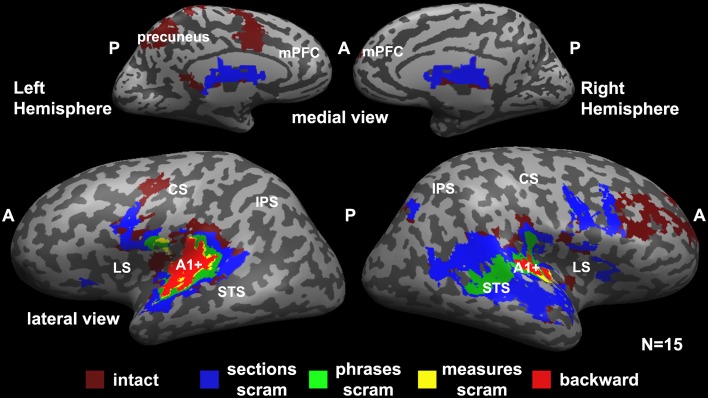
**Hierarchical organization of processing timescales (temporal receptive windows, TRWs)**. Each voxel is colored according to the level of coherent temporal structure that was required to produce significant response reliability (inter-SC) in that voxel across all participants. Red (“backward”), voxels that responded reliably to all stimuli (including the backward stimulus). Yellow (“measures scram”), voxels that responded reliably to all stimuli except the backward stimulus. Green (“phrases scram”), voxels that were reliable only for the phrases, sections, and the intact stimuli. Blue (“sections scram”), voxels that responded reliably only to the sections stimulus and the intact stimulus. Burgundy (“intact”), voxels that responded reliably only to the intact stimulus. A1+, early auditory cortex presumably including primary auditory cortex (A1); LS, lateral sulcus; STS, superior temporal sulcus; CS, central sulcus; IPS, intraparietal sulcus; A, anterior; P, posterior; scram, scrambled.

Figure 2 represents a hierarchy of brain areas, where voxels that were reliable at the lowest hierarchical level responded reliably to all scrambling conditions, and areas at the top of the hierarchy responded reliably to only the intact music. Therefore, a voxel that is colored in red responded reliably to all conditions (backward, measures, phrases, sections, and intact); voxels colored in yellow responded reliably to measures, phrases, sections, and intact stimuli; voxels colored in green responded reliably to phrases, sections, and intact stimuli; voxels colored in blue responded reliably to the sections and intact stimuli; and voxels at the top of the hierarchy responded reliably to only the intact stimuli.

We found a hierarchy of increasingly reliable responses to larger-scale musical structures (Figure 2), starting in the early sensory areas (including primary auditory cortex and adjacent areas of auditory cortex, A1+) and proceeding along the superior temporal gyrus (STG). Early auditory areas showed reliable responses to all stimuli regardless of the timescale of scrambling (Figure 2, red). The extent of reliable activity for the measures condition (Figure 2, yellow) was similar to that for the backward condition. Additional voxels showed reliable responses to phrases, but not to the measures or backward conditions, in the middle STG (mSTG) (Figure 2, green). The sections condition, in turn, evoked reliable responses further up the temporal lobe and the right inferior frontal gyrus (IFG) (Figure 2, blue). Finally, the unaltered, intact condition evoked reliable responses over an even larger region of cortex including the right middle frontal gyrus, dorsal precentral gyrus, and left precuneus (Figure 2, burgundy). The hierarchy was clearer in the left hemisphere, although a weaker topography for temporal structure was observed also in the right hemisphere.

#### Overlap between intact music and intact story

**Figure 3:**
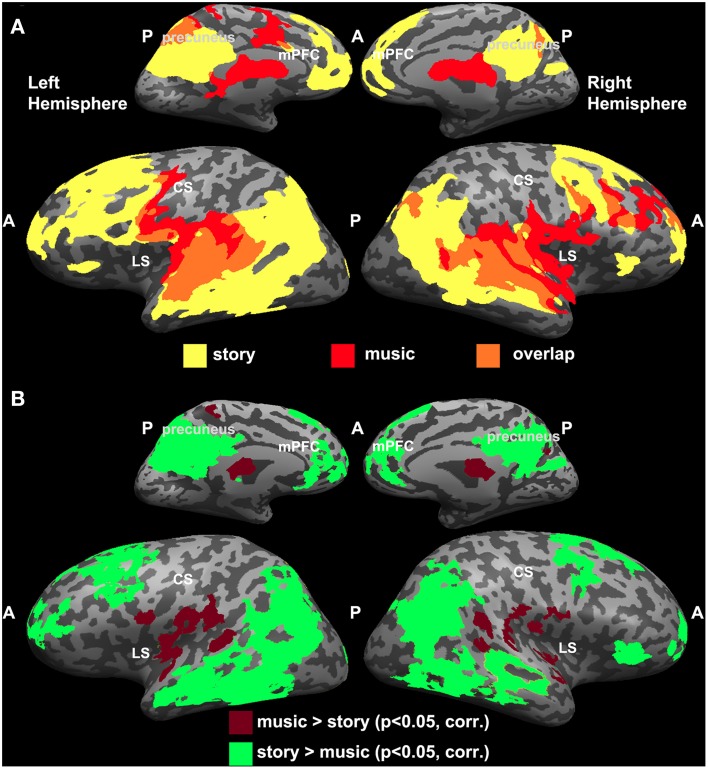
**Comparing reliability between music and speech**. **(A)** Maps of inter-SC for intact story and intact music. Yellow, voxels that responded reliably to the intact story. Red, voxels that responded reliably to the intact music. Orange, voxels that responded reliably to both stimuli. **(B)** Significant differences between intact music and intact story conditions (one-tailed, two-sample *t*-test).

Since the topographic organization observed for music was analogous to the one reported previously for the story experiment (Lerner et al., 2011), we directly compared the results from the two studies (Figure 3). Overlapping regions of reliable responses to intact music and intact story were evident in early auditory areas (A1+) along the STG (Figure 3A). In addition, the story evoked reliable responses in the temporo-parietal junction (TPJ), angular gyrus, IFG (also known as Broca's area), lateral and medial prefrontal areas, and orbitofrontal cortex, whereas reliable responses to music were found in the lateral sulcus, pre-central gyrus, and middle frontal gyrus (Figure 3A). One-tailed, two-sample *t*-tests, applied to Pearson correlation coefficients (see Methods: Inter-SC analysis) revealed statistically significant differences between intact music and intact story conditions (Figure 3B). Specifically, in regions colored in burgundy, inter-SCs were higher for musical stimuli than for speech; regions colored in green showed the opposite effect—inter-SCs were higher for speech than for music in these regions.

#### ROI analysis for music and speech

**Figure 4:**
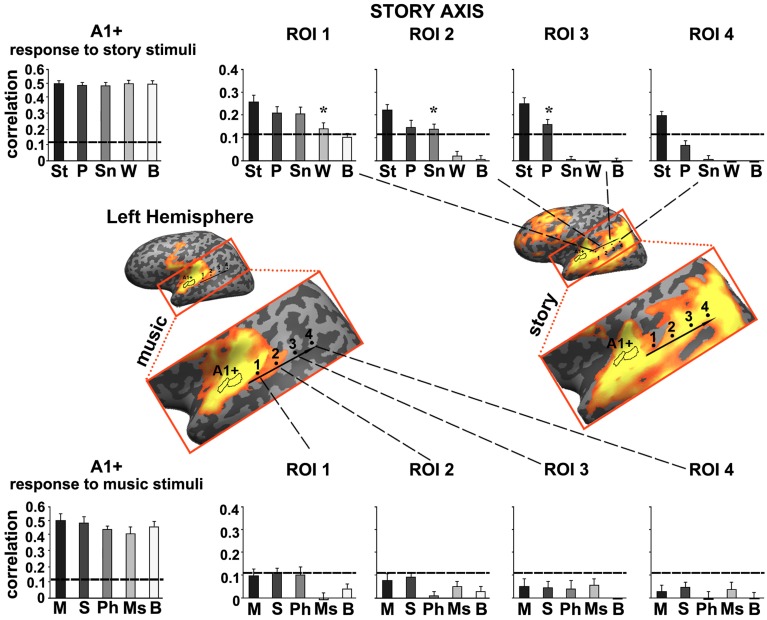
**Response reliability in ROIs defined along a “story axis”—left hemisphere**. Middle row, maps of reliable responses to the intact music (left) and story (right) stimuli. The story axis was manually defined along the map of reliable responses to the intact story (right map). The same axis is superimposed on the map of reliable responses to intact music (left map). Early auditory ROI (A1+) was defined as the set of voxels that correlated with the stimulus audio envelope. ROIs 1–4 were evenly spaced along the axes by manual partitioning the extent of reliable responses into approximately equally sized adjacent sub-regions. Top row, response reliability to story stimuli. Inter-SC is plotted for each of the story stimuli, scrambled at each of several timescales, for each of the ROIs. Horizontal lines indicate thresholds for statistically significant correlations assessed using phase-randomization and false discovery rate procedures (see Methods). Asterisks denote significant differences between reliable responses to scrambled conditions vs. intact condition; ^*^p < 0.05, one-tailed, paired *t*-test. Bottom row, response reliability to music stimuli. Error bars indicate estimated standard error. Abbreviations: St, intact story; P, paragraph; Sn, sentence; W, word; B, backward; M, intact music; S, section; Ph, phrase; Ms, measure.

**Figure 5:**
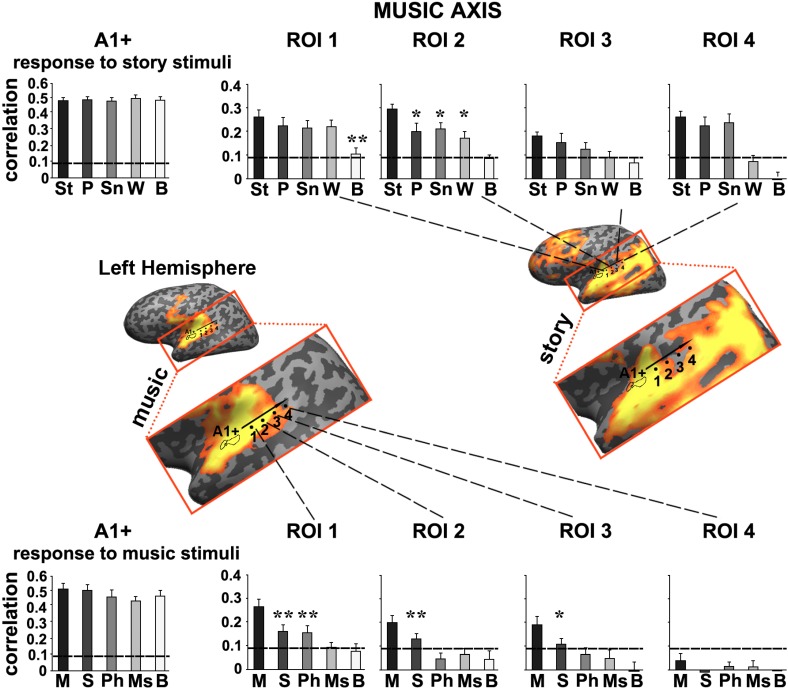
**Response reliability in ROIs defined along a “music axis” in the left hemisphere**. Same format as Figure 4 except that the ROIs were evenly spaced along the music axis, which was manually defined along the map of reliable responses to the intact music stimulus. Error bars indicate estimated standard error. ^*^p < 0.05; ^**^p < 0.005, one-tailed, paired *t*-test. Abbreviations: St, intact story; P, paragraph; Sn, sentence; W, word; B, backward; M, intact music; S, section; Ph, phrase; Ms, measure.

**Figure 6:**
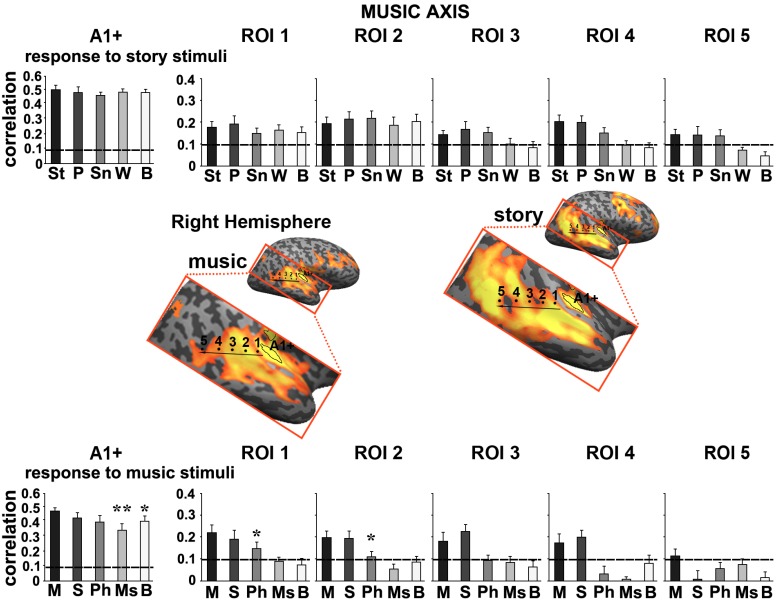
**Response reliability in ROIs defined along a “music axis” in the right hemisphere**. Same format as Figure 5. Abbreviations: St, intact story; P, paragraph; Sn, sentence; W, word; B, backward; M, intact music; S, section; Ph, phrase; Ms, measure.

To further quantify the differences we defined a story axis (Figure 4, left hemisphere) and a music axis in each of the two hemispheres (Figure 5, left hemisphere; Figure 6, right hemisphere), and defined ROIs along each of these axes (see Methods). Early auditory areas (A1+) exhibited high inter-SCs for speech and music, irrespective of the scrambling level. This suggests that the cortical activity in these sensory regions was reliably modulated by instantaneous physical parameters (e.g., timbre, sound amplitude), but processing was largely independent of temporal context. Accordingly, we labeled A1+ as having short TRWs.

Moving along the story axis, we observed a clear topography of temporal structure for speech stimuli in which the reliability of responses to scrambled speech declined gradually (backward, words, sentences, and then paragraphs) from ROI 1 toward ROI 4 (Figure 4, top). In areas with especially long TRWs, such as TPJ and medial prefrontal cortex (mPFC), the cortical activity at each moment depended on over tens of seconds of preceding auditory stimulation. We denoted this region as having long TRWs. ROIs along the story axis did not exhibit reliable responses to music, for any of the timescales (Figure 4, bottom).

Moving along the music axis in each hemisphere, we observed a topography of temporal structure for music stimuli (Figures 5, 6) in which the reliability of responses to scrambled music declined gradually (backward, measures, and then phrases) from ROI 1 toward ROI 4. However, there was evidence for differences in this gradient between the hemispheres. First, early auditory areas (A1+) exhibited more reliable responses in the left hemisphere than the right hemisphere for the backward condition (*p* < 0.03, one-tailed, paired *t*-test). Second, reliable responses to the sections and intact conditions were stronger in the right hemisphere compared to the left hemisphere (ROI 4: intact condition: *p* < 0.02; sections condition: *p* < 2.4E-05, one-tailed, paired *t*-test). The reliability of responses to music dropped for all conditions moving further posteriorly along the STG (see ROIs 4–5). In contrast to the story axis, areas along the music axis did exhibit reliable responses to speech (Figures 5, 6, top panel).

### Inter-subject correlation in subcortical structures for music

**Figure 7:**
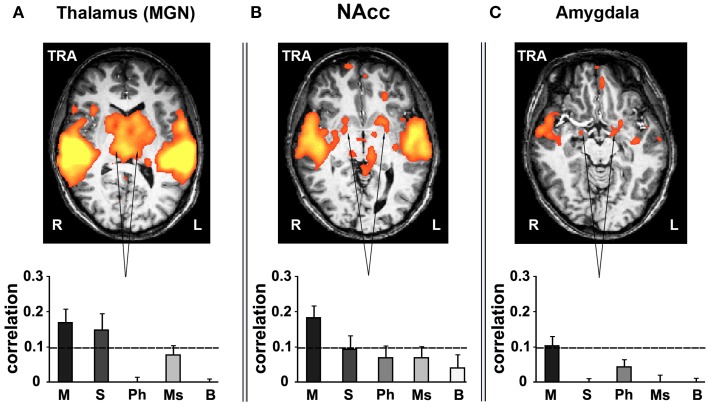
**Inter-SC in the subcortical structures. (A)** thalamus. **(B)** nucleus accumbens (NAcc). **(C)** amygdala. Top row, maps of reliable responses to the intact music stimulus. Bottom row, inter-SC is plotted for each of the music stimuli, scrambled at each of several timescales. Error bars indicate estimated standard error. Abbreviations: TRA, transverse view of brain; R, right hemisphere; L, left hemisphere.

Various subcortical structures associated not only with auditory but also emotion and reward-related processes also exhibited reliable responses to music. Subcortical ROIs were defined anatomically using the Brede database (Nielsen, 2003; http://neuro.imm.dtu.dk/services/brededatabase/WOROI_245.html). The thalamus exhibited reliable responses when presenting listeners with stimuli containing coherent segments over long timescales (Figure 7A). Specifically, measuring inter-SC in the thalamus, we found high correlation values for only the intact and sections music conditions. Likewise, the nucleus accumbens (NAcc, Talairach coordinates: ±17, 9, −2) in the ventral striatum responded reliably only for the intact music condition (Figure 7B). Likewise, the amygdala (Talairach coordinates: ±15, −2, −10) exhibited reliable responses (bilaterally, but more evident in the left hemisphere) only for the intact music condition (Figure 7C). The reliability in the subcortical regions was specific to music; comparably reliable responses in these regions were not found in the story conditions.

## Discussion

An extended musical excerpt and scrambled versions of it were presented to experienced pianists to examine differences in the reliability of brain responses to musical structure at various timescales. In addition, we directly compared the reliability of brain responses to music and speech structures at those timescales. We found that the processing timescale for music became gradually longer toward higher-order brain areas. Such topography of processing timescales parallels the topography found for speech (Lerner et al., 2011). In early auditory cortical areas, brain responses were similarly reliable for all stimuli, including scrambled music at all timescales and backward music, whereas parietal and frontal areas at the apex of the TRW hierarchy responded reliably only when the original, unscrambled piece was heard. In general, our results converge with prior work indicating that increasingly more abstract levels of hierarchical musical structure are encoded as one ascends from early auditory areas to neighboring auditory cortical regions, and then to frontal cortex (Zatorre and Zarate, 2012).

### Long timescales of music processing

Our most surprising finding was the difference between the phrases, sections, and intact music conditions, indicating that some brain areas respond more reliably to global hierarchical structures at timescales far beyond what has been observed in previous behavioral studies on musical coherence. Based on these previous studies (Levinson, 1997; Tillmann et al., 1998; Bigand and Parncutt, 1999; Tillmann and Bigand, 2004), we had expected little or no differences for any timescales longer than a few seconds. Perhaps one reason for why these results contradict previous behavioral studies has to do with the nature of the stimuli—the Brahms features dramatic shifts in texture and expressive dynamics, whereas the Baroque and Classical-style stimuli used in previous studies typically lack such shifts. Along similar lines, Lalitte and Bigand (2006) found that listeners are sensitive to scrambling in contemporary art music.

It might be argued that participants' familiarity with the piece could have been a factor (i.e., expectations due to veridical, long-term memory). However, nearly half of the participants reported having no familiarity with the piece. To further explore this issue, the effect of familiarity was explored by computing correlations between familiarity ratings and inter-SC coefficients (correlation between each participant and the mean of the other participants) for each of the ROIs. These values were then plotted against familiarity ratings, and linear regression was performed. The *R*^2^ values were very small and there was no consistent pattern. It is possible the results were inconclusive due to the small sample size.

Relatively few studies have employed free listening of natural or scrambled music with fMRI, and none have directly examined the issue of long-scale or hierarchical coherence. Levitin and Menon (2003) compared brain responses to unaltered and scrambled versions of classical music excerpts to ascertain whether activation in Brodmann Area 47, a region of the inferior frontal cortex previously identified with structural processing in linguistics, could be associated with music processing. They presented listeners with 23-s-long classical music excerpts and scrambled versions of those excerpts segmented into 250–350 ms fragments and reordered. Similarly, Abrams et al. (2011) compared brain activity patterns of nonmusicians listening to natural and temporally scrambled music and speech. Their original speech/music excerpts were 22–30 s long and the segmentation sizes for both music and speech were on the order of 350 ms. In both the Levitin and Menon (2003) and Abrams et al. (2011) studies, the stimuli timescales were considerably shorter than the ones examined in the current study.

In a follow-up experiment, Abrams et al. (2013) examined brain activity of nonmusicians listening to a 9′35″ amalgam of four symphonies by late-Baroque composer William Boyce. In addition to the original excerpts, participants also listened to spectrally rotated and phase-scrambled versions of the music. Employing a similar inter-SC correlation method, they found reliable responses to the intact music condition in multiple brain areas, including STG, frontal, and parietal cortex, and motor areas. Moreover, similar to this study, they reported reduced reliability for phase-scrambled and spectrally rotated conditions in the right IFG and the intra-parietal sulcus (IPS). Their study supports our findings that these areas are sensitive to the temporal structure of the music. However, our study is the only one to parametrically scramble the music at multiple timescales, and thus the first to reveal the processing timescale topography and the first to reveal that areas at the top of the hierarchy are sensitive to musical structure at minute-long timescales.

Finally, long-timescale coherence evoked reliable responses in subcortical brain structures. The involvement of subcortical structures in processing of emotionally charged auditory stimuli has been extensively investigated previously (Peretz et al., 1998; Blood and Zatorre, 2001; Bigand et al., 2005), demonstrating activity in the amygdala, ventral striatum and midbrain while participants listened to music. In our study these subcortical regions responded reliably only to the intact and sections conditions—consistent with the fact that only the long-timescale conditions are able to carry an emotional component due to stimulus continuity. This supports the findings of Menon and Levitin (2005), which indicated stronger nucleus accumbens responses to intact musical stimuli than scrambled music.

### Lateralization

Previous research on auditory cortical activity has indicated that the left hemisphere specializes in enhanced processing of temporal structure, while the right hemisphere specializes in processing higher frequency resolution, i.e., differences in temporal integration windows (Patel and Balaban, 2001; Zatorre and Belin, 2001; Poeppel, 2003; Boemio et al., 2005; Schönwiesner et al., 2005; Overath et al., 2008; Okamoto et al., 2009). Right-lateralized responses to music are perhaps reflective of the precise discrete-pitch relationships that are essential to melodic processing in a way that has no equivalent in speech (Zatorre et al., 2002; Liégeois-Chauvel et al., 2006).

This lateralization issue is usually discussed in the context of timescales that are tenths to hundredths of a second long, in contrast to our experiment, in which the stimuli were manipulated at timescales on the order of seconds and minutes. However, conceptually our results appear to reflect some of the hypothesized differences between the two hemispheres in the types of musical features that are processed. Early auditory areas exhibited more reliable responses in the left hemisphere than the right hemisphere for the backward condition, for which it is difficult to track coherent melodic lines. In contrast, reliable responses to the sections and intact conditions covered a more extensive region in the right temporal lobe compared to the left hemisphere.

### Musical features

The regions of reliable activation also reflected the processing of specific musical attributes at different scrambling levels. Prior work has shown that rhythmic and metrical processing in music listening are indicated by recruitment of motor areas of the brain that have been linked to beat induction (Chen et al., 2006, 2008). Rhythmic entrainment usually happens within seconds, given a steady, isochronous beat (London, 2012); however, the seeming lack of coordination in this area in all conditions with the exception of the intact excerpt indicated that listeners probably found the tempo contrasts between scrambled segments to be jarring. Perhaps this is not surprising given that the Brahms excerpt has fairly frequent fluctuations in tempo. Unlike pop music, it lacks a strict pulse, and scrambling further reduces a consistent sense of beat.

In the case of melodic processing, reliable responses posterior and anterior to Heschl's gyrus (HG) in the STG for phrase and longer timescales (particularly on the right side) indicated that at a minimum, phrase-length chunks of music were needed for tracking of melodic lines. The neural substrates of melodic processing are found along the STG both anterior and posterior to HG; the posterior auditory cortex is more sensitive to pitch contour while the anterior areas show sensitivity to pitch chroma (i.e., the relative position of a pitch within a scale) (Zatorre and Zarate, 2012).

Harmonic processing and emotional response to music were only evident over longer timescales. Only the sections and intact cases showed reliable activation in the IFG, indicating key recognition and tonal processing had occurred; prior work has shown that areas of the IFG are integral to musical syntax processing (Maess et al., 2001; Tillmann et al., 2003; Koelsch et al., 2005). With regard to emotional response, reliable activation in subcortical structures—thalamus, amygdala, and NAcc—was evident only in the section and intact conditions for thalamus and only intact for the other areas. The ventral striatum (in particular, NAcc), thalamus, and amygdala are all regions that have been associated with emotional response to music in prior studies (Blood and Zatorre, 2001; Brown et al., 2004; Menon and Levitin, 2005; Koelsch et al., 2006; Mitterschiffthaler et al., 2007; Salimpoor et al., 2013).

### Music and speech

Abrams et al. (2011), discussed above in the context of fMRI studies on musical coherence, agree with previous findings that music and speech processing share neural substrates (Besson and Schön, 2001; Maess et al., 2001; Patel, 2003; Koelsch et al., 2005; Carrus et al., 2011), but conclude that temporal structure in the two domains is encoded differently. Another study comparing music and speech by Rogalsky et al. (2011) presented participants with nonsensical sentences, scrambled nonsensical sentences, and novel melodies played back at different rates. They concluded that previous evidence for apparent processing similarity may have been derived from higher-order cognitive mechanisms, such as working memory or cognitive control systems rather than anything specific to music or language per se.

Although our results are consistent with those of Rogalsky et al. (2011) and Tervaniemi et al. (2006) showing dorsomedial regions of the temporal lobe responded more reliably to music and ventrolateral regions responded more reliably to speech, our stimuli differed significantly from either. We explored timescales at all levels of musical form while Rogalsky et al. only examined brain responses to short (~3 s) melodies and Tervaniemi et al. used saxophone sounds that were under 1 s in duration. Moreover, our results are the first to show that the topography of the TRWs for music and speech differ significantly. For example, with the exception of early auditory areas, those regions that exhibited the topography of temporal structure for speech did not respond reliably to music. Consequently the timescale gradients for music and speech overlapped with one another only partially, suggesting that temporally analogous structures—words/measures, sentences/musical phrases, paragraph/sections—are processed differently.

## Author contributions

All authors contributed to the research conceptually, including the experimental design and data interpretation; all authors also contributed to writing and/or revising the paper. MF: designed stimuli, wrote experiment script, acquired fMRI data; YL: acquired fMRI data, analyzed data.

### Conflict of interest statement

The authors declare that the research was conducted in the absence of any commercial or financial relationships that could be construed as a potential conflict of interest.
